# A Natural Composite Extract Restores Skin Barrier Function by Modulating Inflammatory, Hydration, and Redox Pathways

**DOI:** 10.1111/jocd.70613

**Published:** 2025-12-13

**Authors:** Caijun Jin, Youn Jeong Cha, Hee Eun Kim, Yongxun Jin, Xinrui Zhang, Linh Thi Thuy Le, Nguyen Ngan Giang, KeonWoo Choi, Yong‐Hyun Lee, Woon Ha Kim, Jae Hee Choi, Pham Ngoc Chien, Heo Chan Yeong

**Affiliations:** ^1^ Department of Plastic and Reconstructive Surgery, College of Medicine Seoul National University Seoul Republic of Korea; ^2^ Department of Plastic and Reconstructive Surgery Seoul National University Bundang Hospital Seongnam Republic of Korea; ^3^ Zero to Seven Inc. Seoul Republic of Korea; ^4^ Korean Institute of Nonclinical Study Center Seongnam Republic of Korea; ^5^ Department of Plastic and Aesthetic Surgery Peking Union Medical College Hospital Beijing People's Republic of China; ^6^ Department of Biomedical Science, College of Medicine Seoul National University Seoul Republic of Korea; ^7^ Hai Phong University of Medicine and Pharmacy Vietnam; ^8^ Department of Medical Device Development, College of Medicine Seoul National University Seoul Republic of Korea; ^9^ Korean Skin Research Center Seongnam Republic of Korea

**Keywords:** claim substantiation, emulsions, formulation, skin barrier

## Abstract

**Objective:**

This study was to evaluate the effects of a composite extract of *Prunus mume* flower, *Lonicera japonica* flower (honeysuckle), *Chrysanthemum indicum* flower, and *Phyllostachys bambusoides* , which we named PLCP, on skin hydration, inflammation, itching, and oxidative stress in pediatric (NHEK) and adult (HaCaT) keratinocytes.

**Methods:**

LPS was used to simulate the external stimulation for pediatric (NHEK) and adult (HaCaT) keratinocytes. These keratinocytes were treated with different concentrations of PLCP, and the expression of inflammation, itching, and oxidative stress‐related proteins or mRNA was observed through rt‐PCR, ELISA, ROS, and SOD. Meanwhile, rt‐PCR was also used to determine the expression of Hyaluronic acid synthase (HAS) in keratinocytes under PLCP intervention for its moisturizing effects.

**Results:**

PLCP treatment (0.125%–0.500%) significantly suppressed LPS‐stimulated thymic stromal lymphopoietin (TSLP) secretion by up to 40.3% in pediatric cells and 29.9% in adult cells (*p* < 0.05), and reduced endothelin‐1 (ET‐1) mRNA levels by up to 69.5% and 46.0%, respectively (*p* < 0.05). Concurrently, PLCP dose‐dependently upregulated hyaluronic acid synthase isoforms (HAS‐1, HAS‐2, HAS‐3) mRNA expression by 174.8%–254.2% in pediatric keratinocytes and 143.5%–198.9% in adult cells (*p* < 0.05), indicating enhanced hydration potential. Antioxidant assays demonstrated that PLCP at 0.250% decreased reactive oxygen species (ROS) by 55.3% in pediatric and 41.6% in adult cells, while boosting superoxide dismutase (SOD) activity (*p* < 0.05).

**Conclusions:**

Across all endpoints, pediatric keratinocytes exhibited more pronounced responses, suggesting age‐dependent differences in sensitivity. These in vitro findings support PLCP's multifunctional role at anti‐inflammatory, anti‐pruritic, moisturizing, and antioxidant and highlight its promise as a safe, plant‐based alternative for managing pediatric skin disorders.

## 1 Introduction

Skin health is crucial for overall well‐being, with conditions like eczema and atopic dermatitis impacting a significant portion of the population, especially for children [1, 2]. The prevalence of these conditions has been rising, leading to higher morbidity rates and healthcare expenses [3]. Current clinical approaches typically rely on corticosteroids and immunosuppressants, which, although effective, can have adverse effects and may not target the underlying inflammatory processes [4, 5]. Concerns are also growing regarding the long‐term safety of these medications [6]. Therefore, a new potential approach should be explored for skin health and conditions like eczema and atopic dermatitis.

Keratinocytes, the predominant cell type in the epidermis, play a crucial role in maintaining skin homeostasis and reacting to external stressors like inflammation and oxidative stress [7]. In pathological states, these cells display modified gene expression patterns and secrete inflammatory cytokines, such as thymic stromal lymphopoietin (TSLP) [8, 9] and endothelin‐1 (ET‐1) [10, 11], contributing to skin ailments like eczema and atopic dermatitis. The intricate mechanisms governing keratinocyte reactions entail elaborate signaling pathways, notably the nuclear factor kappa B (NF‐κB) [12, 13] and Janus kinase (JAK) pathways, orchestrating inflammatory reactions and oxidative stress adaptation [14].

Despite advancements in understanding keratinocytes' role in skin inflammation and oxidative stress [15, 16, 17], the impacts of specific natural products on these cells are not well studied. PLCP, a composite extract of *Prunus mume* flower, *Lonicera japonica* flower (honeysuckle), *Chrysanthemum indicum* flower, and *Phyllostachys bambusoides* , has been used as an internal medicine in the royal medical treatment for preventing skin damage caused by external environmental factors in ancient Korea. Yet, there is a notable research gap concerning the potential protective effects of PLCP on keratinocytes. This highlights the necessity for further exploration into how PLCP can influence keratinocyte behavior in the realm of skin health and disease management.

*Prunus mume* flower is an ancient medicinal herb that is commonly used in Asian countries with high nutritional ingredients and biological activities. Flavonoids are important functional components in *P. mume* flower with antibacterial and anti‐inflammatory properties [18, 19]. *Lonicera japonica* is a perennial plant in the *Caprifoliaceae* family. As a component of traditional medicine that modulates inflammatory responses, *L. japonica* flower is frequently utilized to treat colds, dysentery, and other ailments [20, 21]. Modern pharmacological studies have shown its broad biological activities, such as antibacterial, anti‐inflammatory, anti‐endotoxin, antiviral, and antipyretic activities [22]. *Chrysanthemum indicum* flower, a perennial plant that has been used as a traditional medicine for more than 2000 years, has isolated and identified 190 chemical constituents from this plant, including flavonoids, terpenoids, and phenylpropanoids, with several pharmacological activities, such as anti‐inflammatory, anti‐oxidation, antipathogenic microorganism, anticancer, immune regulation, and hepatoprotective effects [23]. *Phyllostachys bambusoides* is also used as a traditional medicine with demonstrated effects of anti‐oxidation, free radical scavenging, anti‐inflammatory, liver protection, and ameliorating cognitive deficits with more than 100 chemical compounds, including flavonoids and flavonoid glycosides, volatile components, phenolic acids, polysaccharide, coenzyme Q10, phenylpropanoid, and amino acids [24]. Although the four traditional medicines have been reported for their other anti‐inflammatory and antioxidant effects by many scholars, their application and reports in skin health are rare, so it is necessary to conduct further study. Furthermore, our previous study showed that using our extraction method, the solution demonstrated substantially greater total polyphenol and amino acid contents than those obtained by conventional methods, with the total polyphenol content reaching as high as 497.83 μg GAE/g and total flavonoid content reaching as high as 46.50 μg QE/g [25]. To contextualize our cell‐based findings, we reference an independent QC dataset generated with the same extraction process, showing higher total polyphenol content and superior DPPH antioxidant performance compared with general extraction (Dataset S1).

This study aims to investigate the impact of PLCP on keratinocytes under conditions of inflammation and oxidative stress in both pediatric and adult populations. The research holds significance in advancing our comprehension of skin health and the traditional herb in relief of associated conditions like eczema and atopic dermatitis. Through an emphasis on the protective properties of PLCP, this investigation endeavors to furnish compelling empirical evidence that may underpin its potential utility for strengthening the barrier function of skin against external stimuli and relieving the symptoms of eczema and atopic dermatitis. This study enables a quantitative evaluation of cellular reactions, leading to a comprehensive comprehension of underlying mechanisms. The outcomes of this investigation may advance novel approaches for skin disease prevention and management, underscoring the preventive and therapeutic potential of natural compounds in dermatological care.

## 2 Materials and Methods

### 2.1 Cells and Chemicals

Children's keratinocytes (NHEK, 7‐year‐old donor) were purchased from PromoCell (C‐12011, Sickingenstr, Heidelberg, Germany), and adult keratinocytes (HaCaT) were purchased from AddexBio (T0020001, San Diego, CA, USA). Fetal Bovine Serum (FBS) and Trypsin–EDTA for primary cells were obtained from Gibco‐Life TechnologiesTM (Carlsbad, CA, USA). Human TSLP ELISA Kit was ordered from Invitrogen (Cat: EHTSLP, Waltham, MA, USA).

### 2.2 PLCP Extraction

The extract was prepared by steeping 4 kg of *P. bambusoides* stems, 125 g of *L. japonica* flowers, 12.5 g of *P. mume* flowers, 125 g of *C. indicum* flowers, and 500 g of *P. bambusoides* leaves in a pressure vessel with 25 kg of 10% 1,2‐hexanediol (Symrise, Germany) for 1 week. After the 1‐week steeping, the pressure inside the bamboo was reduced to 0–0.1 bar for 24 h using a pressure reducing pump (A‐1000S, EYELA, Japan) to remove the gas. After the reduction, the inside of the extractor was pressurized to 2–3 bar and maintained for 24 h. This depressurization and pressurization process was repeated for 1 week to recover the bamboo extract, which was filtered under reduced pressure using a prefilter (AF‐101H, HYUNDAI MICRO, Korea), aged at room temperature for 1 week, and then filtered using a 0.45 μm membrane (Cartridge Filters 0.45 μm pore size, hydrophilic PVDF, 90 mm membrane, Durapore, USA). The produced *P. mume* flower, *L. japonica* flower (honeysuckle), *C. indicum* flower, and *P. bambusoides* complex extract was named PLCP. The present batch followed this vacuum energy extraction workflow. Batch‐level QC from an independent laboratory indicates elevated total polyphenols and improved DPPH IC₅₀ relative to general extraction (Table S1), supporting chemical plausibility for the observed bioactivities. In addition, we have included the compositional data of the PLCP extract, which specifies the percentage range of each ingredient (Table S2) and confirms the botanical composition used in this study.

### 2.3 Cell Culture and Treatment Conditions

NHEK and adult HaCaT were seeded at 2.0 × 10^5^ cells/well in 6‐well plates and cultured in DMEM with 10% FBS and 1% penicillin/streptomycin (PS) for 24 h at 37°C in 5% CO_2_. Then, cells were washed with phosphate buffered saline (PBS) and incubated in serum‐free DMEM for 24 h to induce starvation. Medium was then replaced with DMEM with 10% FBS and 1% PS containing LPS (5 μg/mL) in all groups except the untreated control for subsequent experiments. Meanwhile, cells were treated with different concentrations of PLCP, dexamethasone (DEX), retinoic acid (RA), ascorbic acid (AA), and quercetin (QCT). After the indicated incubation time, the cell pellets were collected to isolate total mRNA or protein for further experiments.

### 2.4 WST‐8 Cell Viability Assay

Cell viability was evaluated using the WST‐8 assay. HaCaT cells were seeded at a density of 1 × 10^4^ cells per well in 96‐well plates and incubated for 24 h. The cells were then treated with the PLCP extract at concentrations of 0.0625%, 0.125%, 0.25%, 0.5%, and 1% for an additional 24 h. After treatment, WST‐8 reagent was added to each well, and the plates were incubated for 1 h at 37°C in a 5% CO_2_ incubator. Absorbance was measured at 450 nm using a microplate spectrophotometer (SPECTRAmax, Molecular Devices, USA).

### 2.5 Evaluation of TSLP Content

The ELISA assay was performed using a human TSLP ELISA kit under the manufacturer's guideline. Briefly, the cell medium was collected. Total protein levels in the homogenates were examined by the PierceTM BCA Protein Assay Kit (Thermo Scientific, USA). Absorbance was measured at 450 nm using a microplate reader BioTek Epoch 2 microplate spectrophotometer (BioTek Instruments, VT, USA), and TSLP levels were quantified accordingly.

### 2.6 Total RNA Isolation and RT‐PCR

Total RNA extraction was conducted utilizing RNAiso Plus reagent (Takara Bio, Shiga, Japan), meticulously following the manufacturer's guidelines. Approximately 1 μg of purified total RNA was then subjected to complementary DNA (cDNA) synthesis using the RevertAid First Strand cDNA Synthesis Kit (Thermo Fisher Scientific, MA, United States). Specific primer pairs that analyzed the expression of *ET‐1*, *HAS‐1*, *HAS‐2*, *HAS‐3*, and *GAPDH* are shown in Table 1.

**TABLE 1:** The qPCR primer sequences of this study.

Type	Gene description	Sequences (5′ → 3)
Human	Hyaluronic acid synthase 1 (*HAS‐1*)	F: CTGCGATACTGGGTAGCCTTCA R: CCAGGAACTTCTGGTTGTACCAG
	Hyaluronic acid synthase 2 (*HAS‐2*)	F: GTCATGTACACAGCCTTCAGAGC R: ACAGATGAGGCTGGGTCAAGCA
	Hyaluronic acid synthase 3 (*HAS‐3*)	F: CTTAAGGGTTGCTTGGC R: GTTCGTGGGAGATGAAGGAA
	Endothelin‐1 (*ET‐1*)	F: TCTGCTGGTTCCTGACGTGC R: GGAATGTTTTGAACGAGGACG
	Glyceraldehyde‐3‐phosphate dehydrogenase (*GAPDH*)	F: GTGGTCTCCTCTGACTTCAACA R: CTCTTCCTCTTGTGCTCTGCT

For quantitative PCR, TB Green Premix Ex Taq II (Takara Bio, Shiga, Japan) was used on the QuantStudio 3 Realtime PCR (Thermo Fisher Scientific, MA, United States) with the following PCR conditions: initial denaturation at 95°C for 30 s, followed by 45 cycles of amplification at 95°C for 5 s and 60°C for 34 s. Relative mRNA expression values were determined using the comparative CT method, the 2^−ΔΔCT^ method.

### 2.7 ROS Assay

Cells were washed with PBS, and 100 μL of 20 μM 2′,7′‐dichlorodihydrofluorescein diacetate (DCF‐DA) solution was added. Cells were incubated for 45 min at 37°C in 5% CO_2_. The DCF‐DA solution was prepared in phenol red‐free, serum‐free DMEM (high glucose, with HEPES and 1% PS) to prevent interference during fluorescence measurement. After incubation, cells were washed with PBS, and 100 μL of phenol red‐ and serum‐free DMEM was added. Fluorescence intensity was measured using a Multi‐Mode Microplate Reader (Molecular Devices, USA) at 485 nm excitation and 535 nm emission.

### 2.8 SOD Assay

20 μL of each culture supernatant was transferred to a new 96‐well plate, and 200 μL of WST working solution of SOD assay kit was added. Subsequently, 20 μL of xanthine oxidase enzyme solution was added to each well, and the mixture was incubated for 20 min at 37°C in 5% CO_2_. Absorbance was measured at 450 nm using a Multi‐Mode Microplate Reader (Molecular Devices, USA) for analysis.

### 2.9 Statistical Analysis

All experimental data were collected through measurements and presented as mean ± standard error of the mean (SEM). Statistical analysis was performed using SPSS Package Program version 20 (IBM, USA), with group differences evaluated using one‐way ANOVA. A *p*‐value < 0.05 was considered statistically significant, with significance levels denoted as follows: *p < 0.05, **p < 0.01, ***p < 0.001.

The TSLP activity inhibition rate, ET‐1 mRNA expression inhibition rate, HAS mRNA expression increase rate, ROS scavenging ability (%)*cellular ROS assay and ROS scavenging ability (%)*SOD assay are calculated as follows:

$$\text{TSLP activity inhibition rate}\;\left(\%\right)=\frac{\text{TSLP concentration in negative control group}-\text{TSLP concentration in test group}}{\text{TSLP concentration in negative control group}}$$

$$\mathrm{ET}-1\;\text{mRNA expression inhibition rate}\;\left(\%\right)=\frac{\text{mRNA expression in negative control group}-\text{mRNA expression in test group}}{\text{mRNA expression in negative control group}}$$

$$\begin{array}{c} \mathrm{ROS}\;\text{scavenging ability}\;\left(\%\right)*\text{cellular}\;\mathrm{ROS}\;\text{assay}\\ =\frac{\text{fluorescence intensity of negative control group}-\text{fluorescence intensity of test group}}{\text{fluorescence intensity of negative control group}} \end{array}$$

$$\begin{array}{c} \mathrm{ROS}\;\text{scavenging ability}\;\left(\%\right)*\mathrm{SOD}\;\text{assay}\\ =\frac{\text{aborbance of negative control group}-\text{aborbance of test group}}{\text{aborbance of negative control group}} \end{array}$$

$$\mathrm{HAS}\;\text{mRNA expression increase rate}\;\left(\%\right)=\frac{\text{mRNA expression in test group}-\text{mRNA expression in untreated group}}{\text{mRNA expression in untreated group}}$$

## 3 Result

### 3.1 Scheme and Experiment Setup for Evaluating the Effect on the Skin Barrier Biomarker in Keratinocytes Induced by PCLP

**FIGURE 1:**
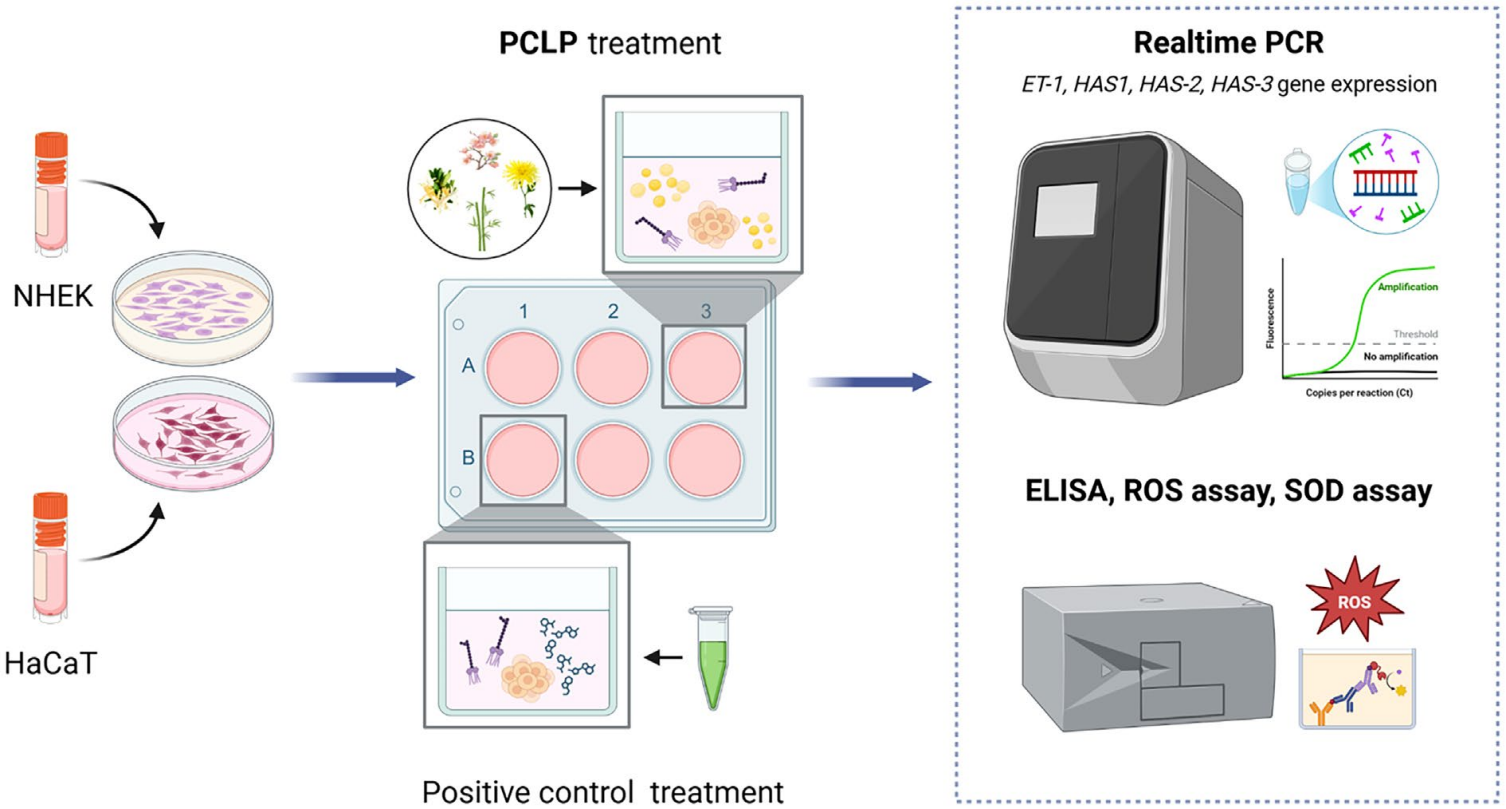
The scheme of an in vitro experiment to assess the efficiency of the PCLP compound in inducing biomarkers of hydration and structure in keratinocytes.

The Child‐derived keratinocytes (NHEK, 7‐year‐old donor) and adult keratinocytes were treated with the PCLP at different concentrations (0.125%, 0.250%, and 0.500%) as depicted in Figure 1. After treatment, we collected the cells and used them to isolate total RNA and protein in order to assess the expression of several biomarkers associated with moisture, inflammation, and oxidative improvement.

### 3.2 Cytotoxicity of the PCLP Extract in HaCaT Cells

**FIGURE 2:**
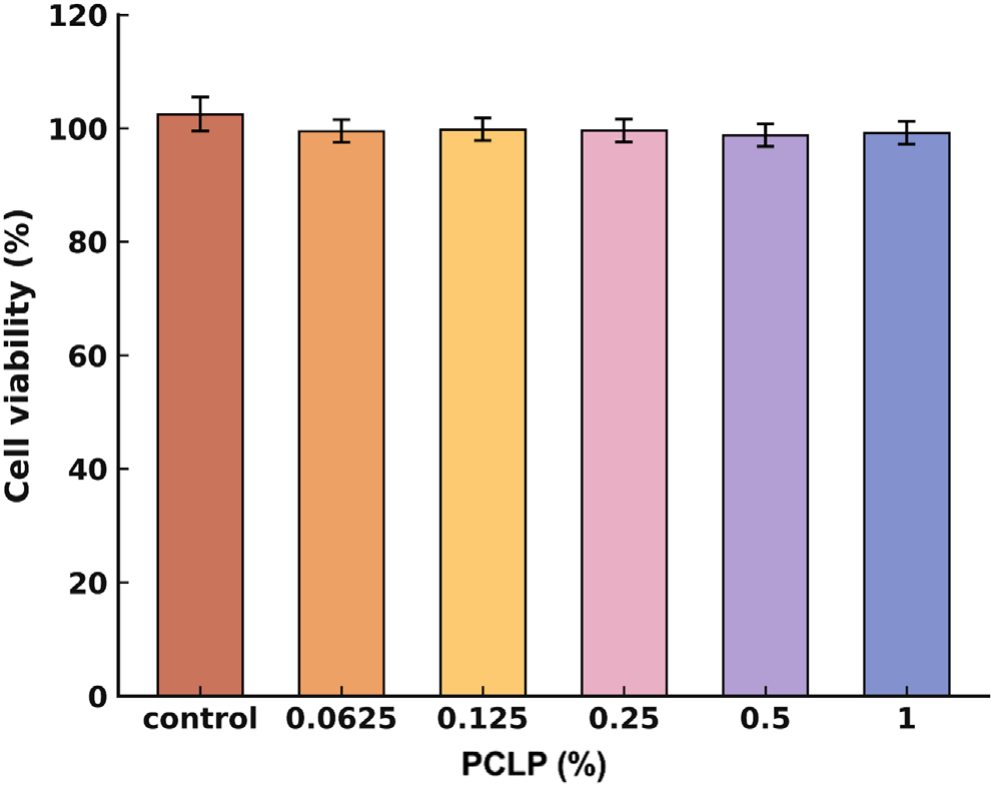
The cytotoxicity of PCLP compound in HaCaT cells. The cell viability was measured by WST‐8 assay. HaCaT cells were incubated with or without PCLP compound at indicated doses for 24 h.

No cytotoxicity was detected in either the control group or any of the groups treated with different concentrations of the PLCP extract. Therefore, subsequent experiments to evaluate the protective effects of the PLCP extract were conducted using concentrations below 1% (Figure 2).

### 3.3 Effect of PCLP on Anti‐Inflammation

**FIGURE 3:**
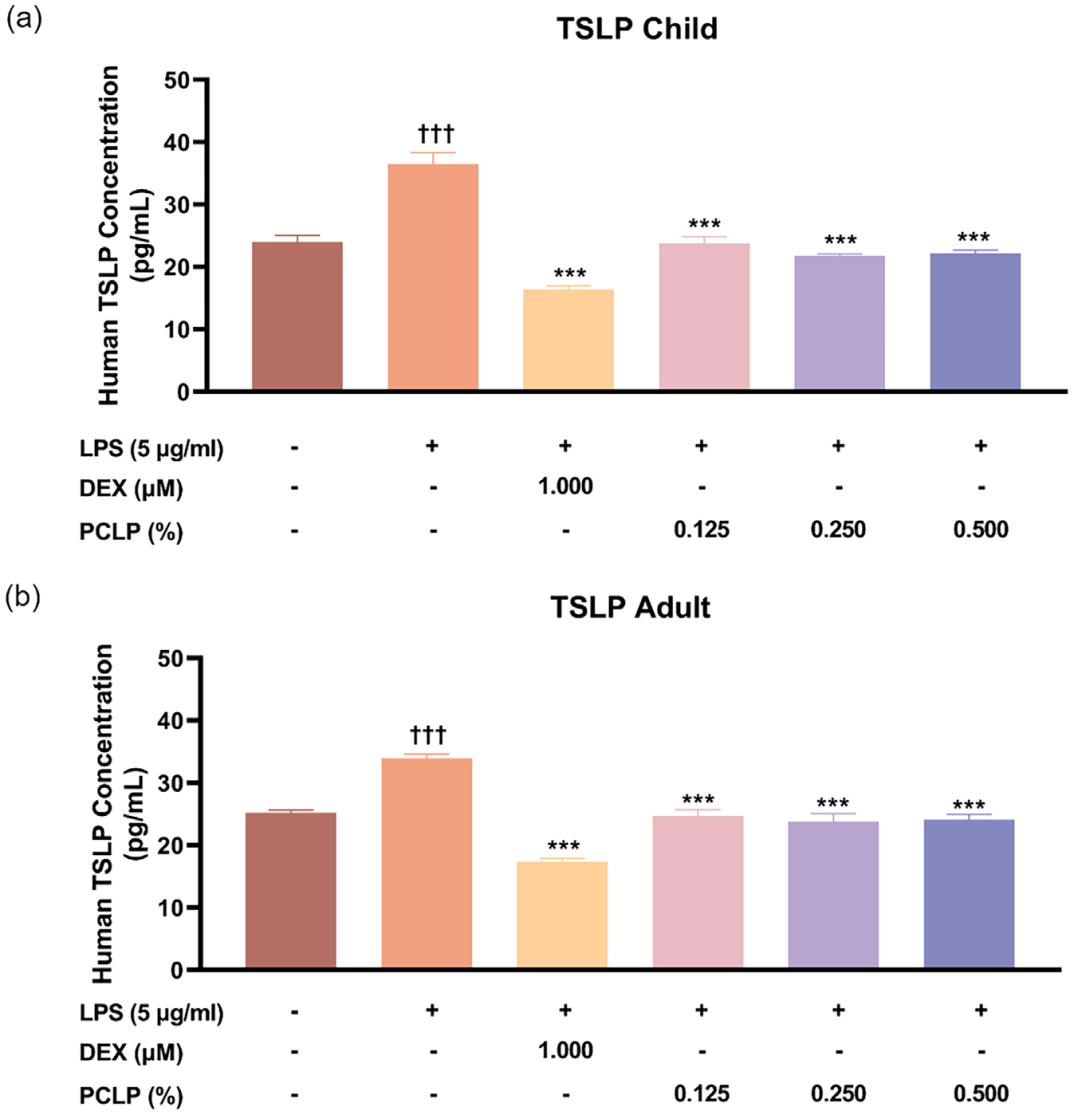
The effect of PCLP on the concentration of the TSLP. The results were obtained using the ELISA method for evaluating the protein expression from LPS‐induced NHEK (*n* = 3) or HaCaT (*n* = 3) after treatment with the indicated concentration of PCLP. DEX, dexamethasone; LPS, lipopolysaccharide; a natural composite extract of *Prunus mume* flower, *Lonicera japonica* flower (honeysuckle), *Chrysanthemum indicum* flower, and *Phyllostachys bambusoides*; TSLP, thymic stromal lymphopoietin. *p < 0.05, **p < 0.01, ***p < 0.001 compared with negative control group (LPS‐induced but untreated). ^†^ *p* < 0.05, ^††^ *p* < 0.01, ^†††^ *p* < 0.001 compared with untreated control.

In NHEK, TSLP expression in the LPS‐treated group (LPS+) increased by 34.31% compared to the untreated control (LPS−) (*p* < 0.05). Treatment with PCLP significantly reduced TSLP levels by 35.01%, 40.34%, and 39.18% at concentrations of 0.125%, 0.250%, and 0.500%, respectively, relative to the LPS+ group (*p* < 0.05, Figure 3a). In HaCaT, TSLP expression in the LPS+ group increased by 25.57% compared to the untreated group (*p* < 0.05), while PCLP treatment groups reduced TSLP levels by 26.98%, 29.85%, and 28.82% at the respective concentrations (*p* < 0.05, Figure 3b). These findings indicate that PCLP significantly inhibits LPS‐induced TSLP expression in both pediatric and adult keratinocytes, suggesting anti‐inflammatory potential.

### 3.4 Effect of PLCP on Itching Improvement

**FIGURE 4:**
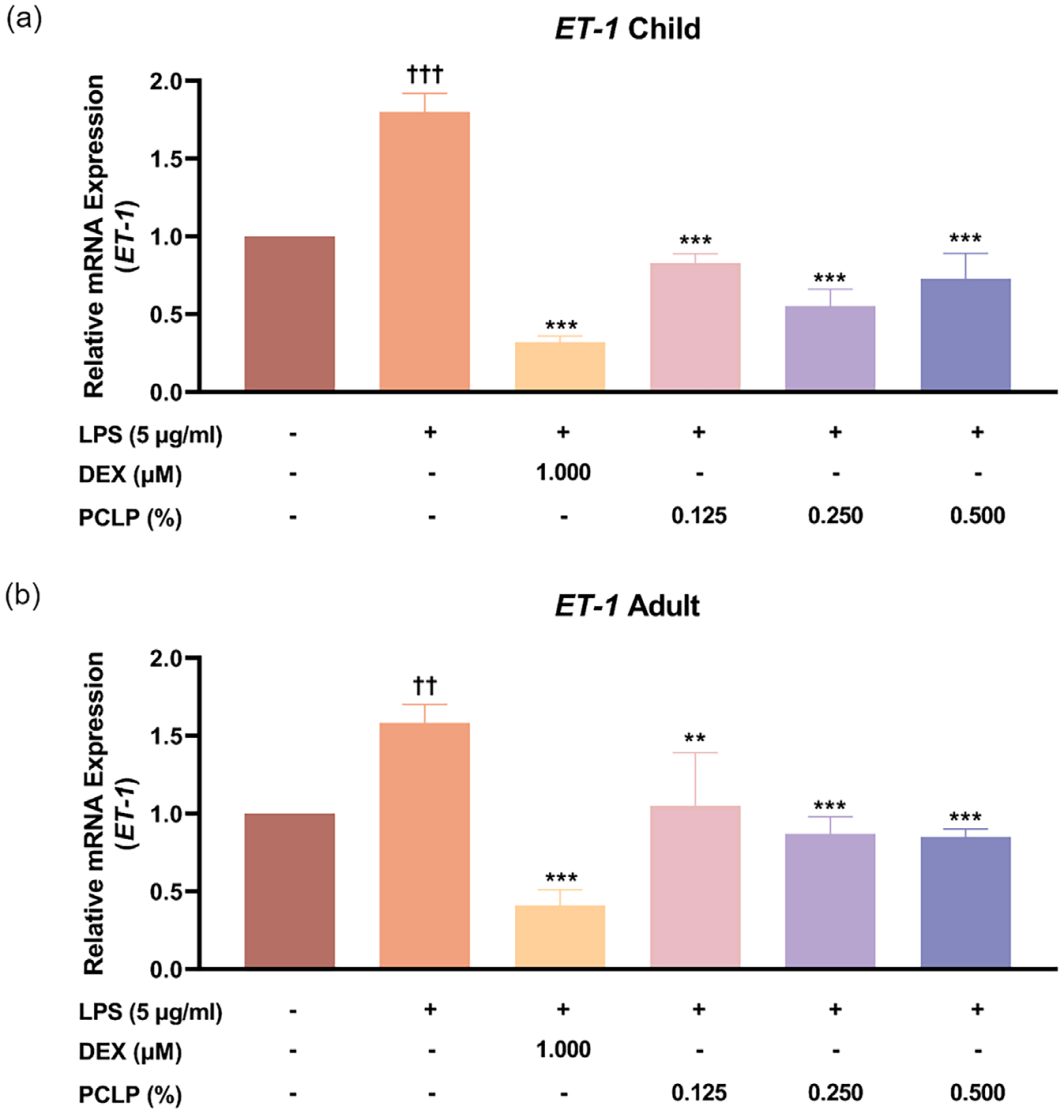
The effect of PCLP on the expression of the ET‐1 gene. The results were obtained using the RT‐PCR method for evaluating the mRNA expression from LPS‐induced NHEK (*n* = 3) or HaCaT (*n* = 3) after treatment with the indicated concentration of PCLP. DEX, dexamethasone; ET‐1, endothelin‐1; LPS, lipopolysaccharide; PCLP, a natural composite extract of *Prunus mume* flower, *Lonicera japonica* flower (honeysuckle), *Chrysanthemum indicum* flower, and *Phyllostachys bambusoides*. *p < 0.05, **p < 0.01, ***p < 0.001 compared with negative control group (LPS‐induced but untreated). ^†^ *p* < 0.05, ^††^ *p* < 0.01, ^†††^ *p* < 0.001 compared with untreated control.

In NHEK, ET‐1 mRNA expression in the LPS‐treated group (LPS+) was significantly elevated by 44.50% compared to the untreated group (LPS−) (*p* < 0.05). PCLP treatment significantly reduced ET‐1 mRNA expression by 53.93%, 69.47%, and 59.39% at concentrations of 0.125%, 0.250%, and 0.500%, respectively, compared to the LPS+ group (*p* < 0.05, Figure 4a). In HaCaT, LPS exposure increased ET‐1 mRNA levels by 36.79% compared to the untreated group (*p* < 0.05), while PCLP decreased expression by 33.68%, 45.00%, and 46.03% at the same concentrations (*p* < 0.05, Figure 4b). These results indicate that PCLP effectively suppresses LPS‐induced ET‐1 mRNA expression in both pediatric and adult keratinocytes, supporting its potential to modulate ET‐1–associated inflammatory and itching responses in skin cells.

### 3.5 Effect of the PCLP on Skin Moisturizing Structure

**FIGURE 5:**
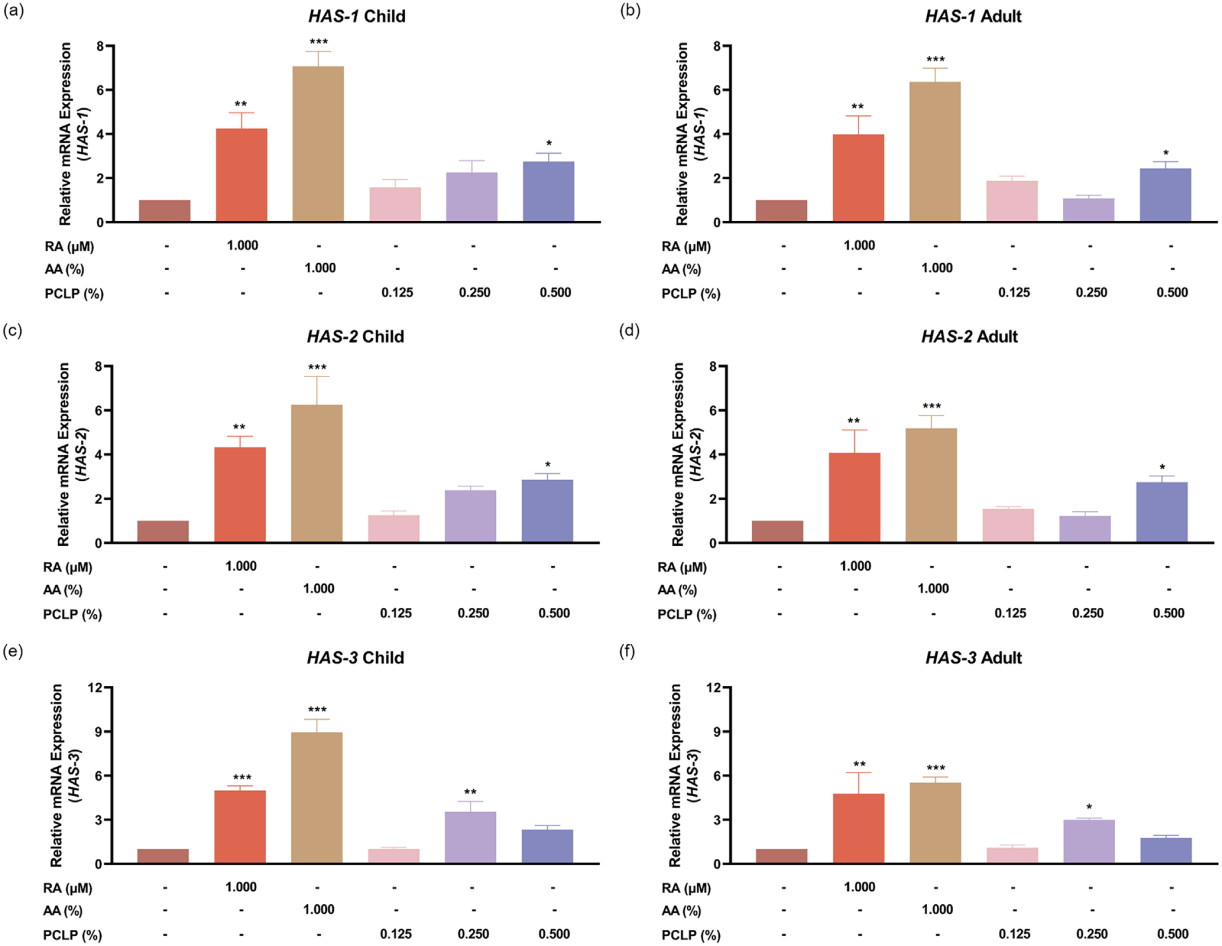
The effect of PCLP on the expression of the HAS‐1 (a, b), HAS‐2 (c, d), and HAS‐3 (e, f) gene. The results were obtained using the RT‐PCR method for evaluating the mRNA expression from NHEK (*n* = 3) or HaCaT (*n* = 3) after treatment with the indicated concentration of PCLP. AA, Ascorbic acid; HAS‐1, hyaluronic acid synthase 1; HAS‐2, hyaluronic acid synthase 2; HAS‐3, hyaluronic acid synthase 3; PCLP, a natural composite extract of *Prunus mume* flower, *Lonicera japonica* flower (honeysuckle), *Chrysanthemum indicum* flower, and *Phyllostachys bambusoides*; RA, Retinoic acid. *p < 0.05, **p < 0.01, ***p < 0.001 compared with the untreated control.

In NHEK, HAS‐1 mRNA expression in the PCLP treatment group significantly increased by 174.77% at a concentration of 0.500% compared to the untreated control (*p* < 0.05), with additional increases of 59.25% and 126.48% observed at 0.125% and 0.250%, respectively (Figure 5a). In HaCaT, HAS‐1 mRNA expression was significantly elevated by 143.53% at 0.500% (*p* < 0.05), and increased by 88.40% and 8.01% at 0.125% and 0.250%, respectively (Figure 5b).

Furthermore, the level of HAS‐2 mRNA expression in the PCLP treatment group significantly increased by 186.32% at 0.500% compared to the untreated control (*p* < 0.05), with additional increases of 25.93% and 139.43% observed at 0.125% and 0.250%, respectively, in NHEK (Figure 5c). While in HaCaT, HAS‐2 mRNA expression was significantly upregulated by 173.62% at 0.500% (*p* < 0.05), and elevated by 53.70% and 23.45% at 0.125% and 0.250%, respectively (Figure 5d).

Additionally, in NHEK, HAS‐3 mRNA expression in the PCLP treatment group significantly increased by 254.17% at 0.250% compared to the untreated control (*p* < 0.05), with additional increases of 1.69% and 132.63% observed at 0.125% and 0.500%, respectively (Figure 5e). In HaCaT, HAS‐3 expression was significantly elevated by 198.86% at 0.250% (*p* < 0.05), along with increases of 9.90% and 78.37% at 0.125% and 0.500%, respectively (Figure 5f).

These results demonstrate that PCLP upregulates HAS‐1, HAS‐2, and HAS‐3 mRNA expression in both pediatric and adult keratinocytes, with a concentration‐dependent effect, particularly prominent in the pediatric group. Given the role of HAS‐1, HAS‐2, and HAS‐3 in hyaluronic acid synthesis, these suggest potential for enhancing skin hydration and barrier integrity.

### 3.6 Effect of Antioxidant From PCLP

**FIGURE 6:**
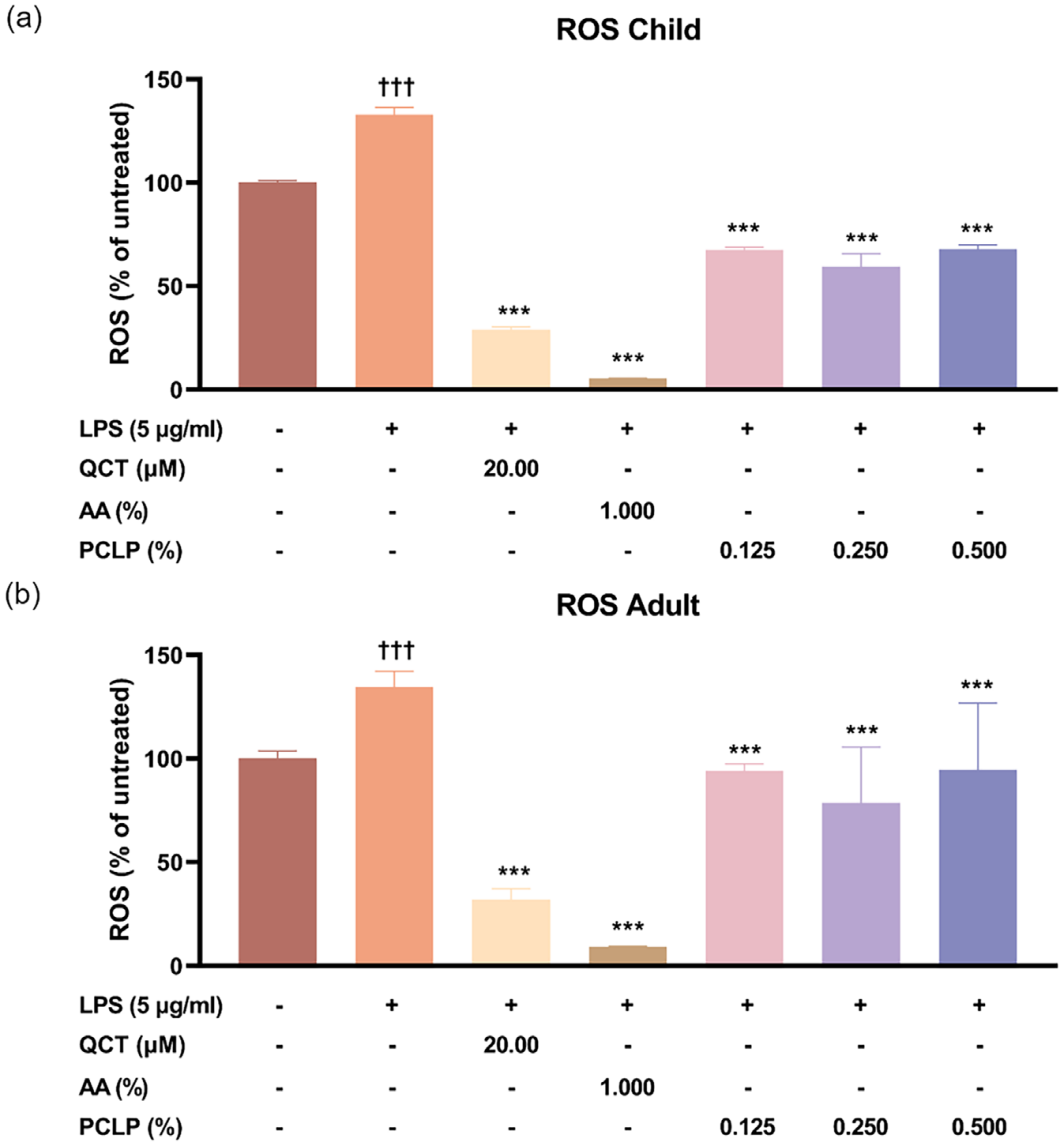
The ability of PCLP on the intracellular ROS scavenging under LPS‐induced condition. The results were obtained using the cellular ROS assay method for evaluating the intracellular ROS scavenging ability from LPS‐induced NHEK (*n* = 3) or HaCaT (*n* = 3) after treatment with the indicated concentration of PCLP. AA, ascorbic acid; LPS, lipopolysaccharide; QCT, quercetin; PCLP, a natural composite extract of *Prunus mume* flower, *Lonicera japonica* flower (honeysuckle), *Chrysanthemum indicum* flower, and *Phyllostachys bambusoides*; ROS, reactive oxygen species. *p < 0.05, **p < 0.01, ***p < 0.001 compared with negative control group (LPS‐induced but untreated). ^†^ *p* < 0.05, ^††^ *p* < 0.01, ^†††^ *p* < 0.001 compared with untreated control.

For ROS assay, in NHEK, ROS levels in the LPS‐treated group (LPS+) increased by 24.77% compared to the untreated control (LPS−) (*p* < 0.05). PCLP treatment significantly reduced ROS levels by 49.14%, 55.32%, and 49.01% at concentrations of 0.125%, 0.250%, and 0.500%, respectively, relative to the LPS+ group (*p* < 0.05, Figure 6a). In HaCaT, LPS treatment increased ROS levels by 25.73% (*p* < 0.05), while PCLP decreased ROS by 30.16%, 41.62%, and 29.78% at the same respective concentrations (*p* < 0.05, Figure 6b).

**FIGURE 7:**
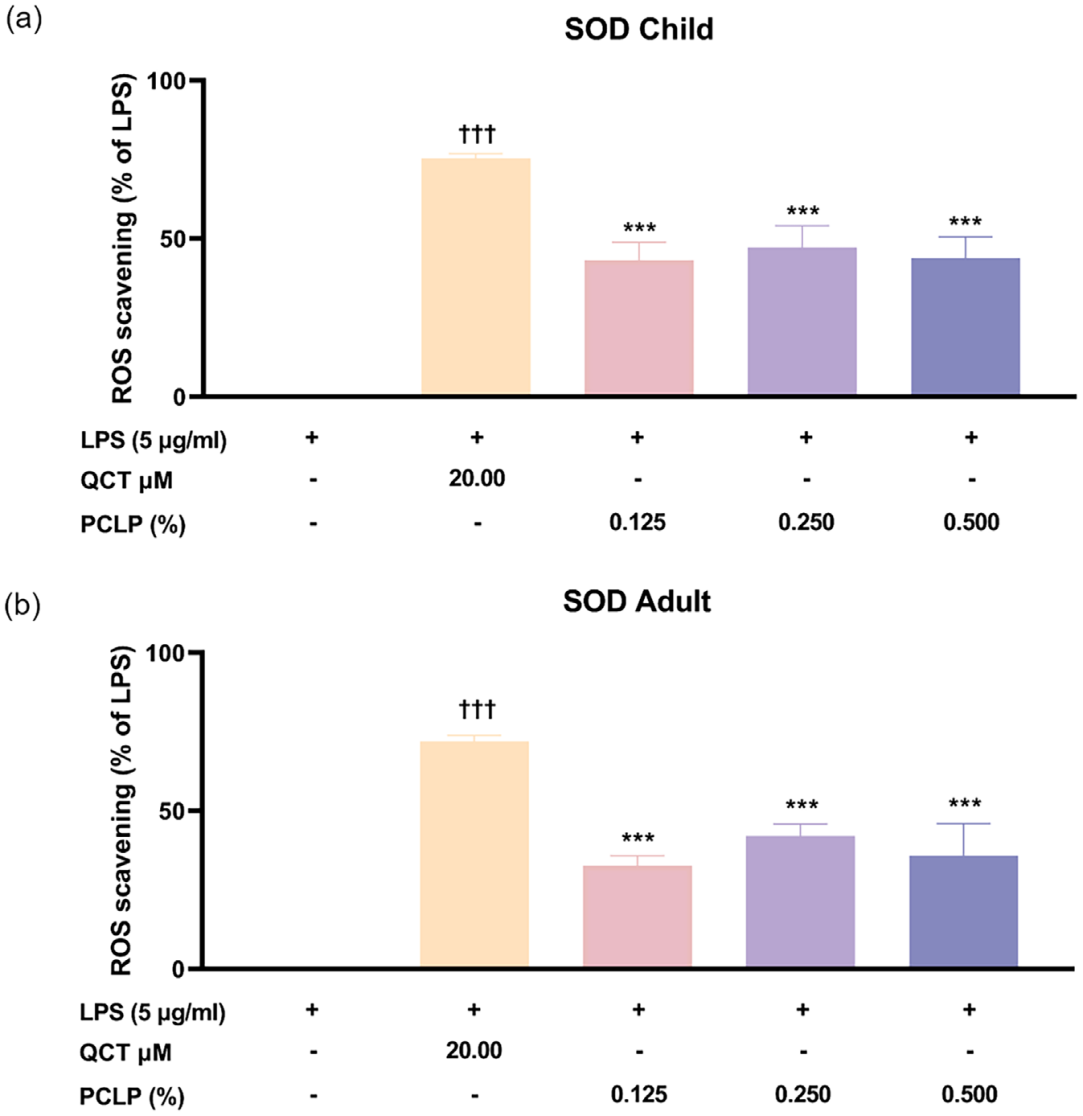
The ability of PCLP on the ROS scavenging under LPS‐induced condition. The results were obtained using the SOD assay method for evaluating the ROS scavenging ability from LPS‐induced NHEK (*n* = 3) or HaCaT (*n* = 3) after treatment with the indicated concentration of PCLP. AA, ascorbic acid; LPS, lipopolysaccharide; QCT, quercetin; PCLP, a natural composite extract of *Prunus mume* flower, *Lonicera japonica* flower (honeysuckle), *Chrysanthemum indicum* flower, and *Phyllostachys bambusoides*; ROS, reactive oxygen species. *p < 0.05, **p < 0.01, ***p < 0.001 compared with negative control group (LPS‐induced but untreated). ^†^ *p* < 0.05, ^††^ *p* < 0.01, ^†††^ *p* < 0.001 compared with untreated control.

Similarly to ROS assay, the antioxidant effect of the PCLP was measured by SOD assay. The result showed that in NHEK, PCLP significantly reduced ROS levels by 43.09%, 47.17%, and 43.69% at concentrations of 0.125%, 0.250%, and 0.500%, respectively, compared to the LPS+ group (*p* < 0.05, Figure 7a). In HaCaT, PCLP treatment decreased ROS by 32.60%, 42.06%, and 35.79% at the corresponding concentrations (*p* < 0.05, Figure 7b).

These results indicate that PCLP effectively attenuates LPS‐induced oxidative stress in both pediatric and adult keratinocytes, with the most pronounced antioxidant effect observed at 0.250%, supporting its potential as a therapeutic agent for skin protection.

## 4 Discussion

**FIGURE 8:**
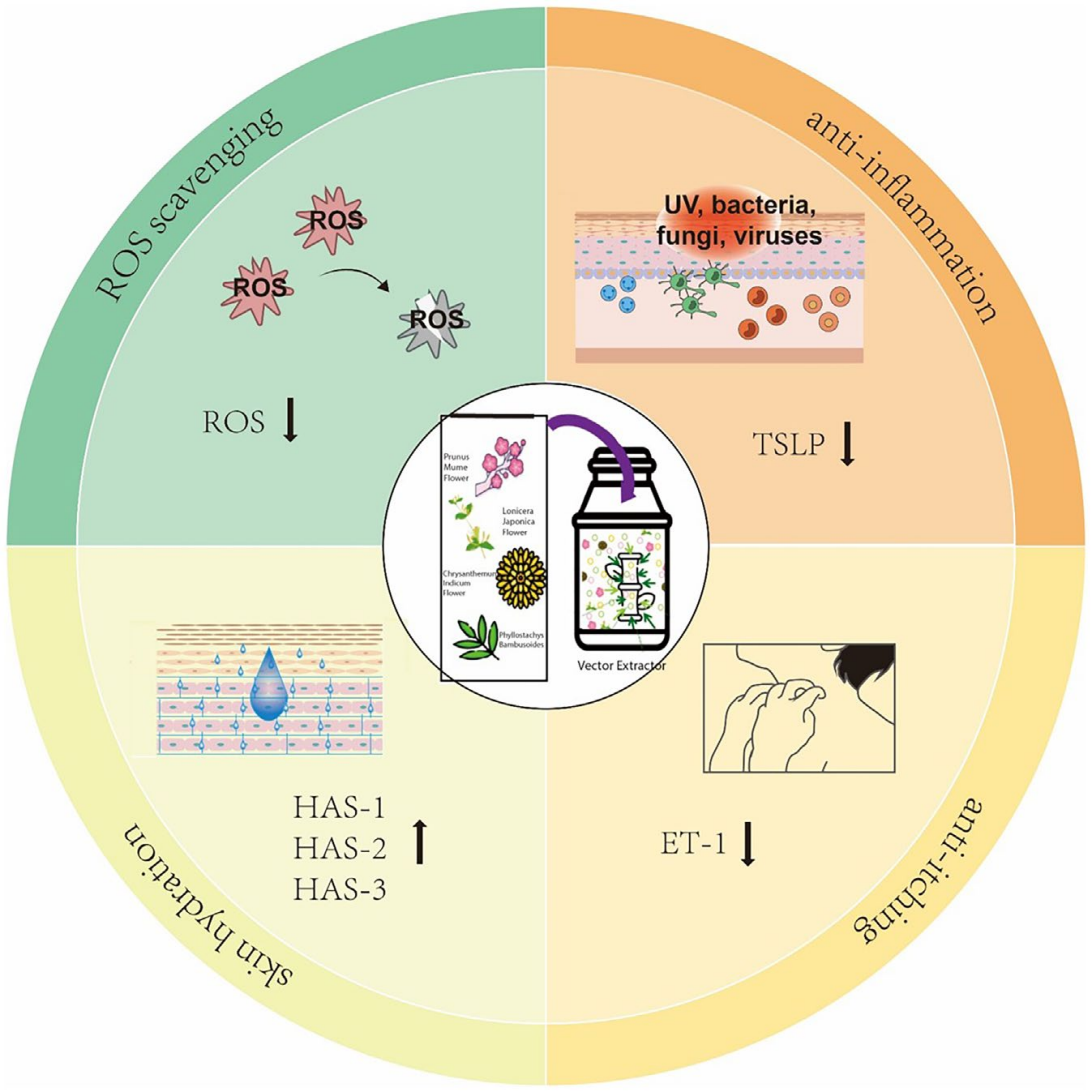
The role of PCLP in skin health.

The epidermis, as the body's first natural line of defense against the external environment, can effectively protect against mechanical, physical, chemical, and biological external stimuli. Keratinocytes account for 90% of epidermal cells, and their main function is to resist damage to the body caused by environmental factors such as heat, ultraviolet rays, dehydration, pathogenic bacteria, fungi, parasites, and viruses. In other words, keratinocytes play a vital role in regulating inflammation, moisture, and oxidative stress. In this study, we systematically evaluated the potential protective effect of PCLP in skin health through multiple experiments for both child and adult keratinocytes. In vitro models using LPS stimulation were applied to mimic inflammatory skin conditions, allowing a comparative assessment of age‐specific responses to the test compound across multiple biological endpoints. We found that PCLP inhibited cytokine expression and played a protective role in the development of LPS‐induced oxidative stress and inflammatory responses, supporting its potential role as a skin protectant (Figure 8).

Thymic stromal lymphopoietin (TSLP) is a key epithelial‐derived cytokine implicated in the initiation of type 2 inflammation and pruritus, especially in disorders such as atopic dermatitis [26, 27, 28]. TSLP promotes the Th2 immune response, which leads to a chronic Th2 inflammatory response and stimulation of the sensory neurons, which ultimately induces the itching symptom [29, 30]. LPS‐induced TSLP elevation observed in this study is consistent with previous findings that TLR4 activation enhances TSLP transcription in keratinocytes through NF‐κB and MAPK signaling [31, 32, 33]. Our research showed that PCLP significantly suppressed TSLP levels in both NHEK and HaCaT cells, suggesting it modulates upstream signaling pathways linked to pruritic and inflammatory responses.

Additionally, Endothelin‐1 (ET‐1) is another critical itch mediator, directly activating sensory neurons and perpetuating local inflammation via endothelin receptor signaling in keratinocytes [34, 35]. The LPS‐induced upregulation of *ET‐1* mRNA confirmed the model's capacity to reproduce pruritic signaling. The significant downregulation of ET‐1 expression by PCLP, particularly at 0.250% and 0.500%, indicates a potential mechanism for itch suppression, possibly via inhibition of ERK1/2 or PLC‐dependent transcriptional pathways [36, 37].

These findings align with previous reports showing that anti‐inflammatory skin actives—especially those derived from natural extracts—can effectively reduce TSLP and ET‐1 levels, thereby alleviating itch and inflammation in vitro and in vivo [38, 39]. The dual inhibition of both TSLP and ET‐1 by PCLP highlights its promise as a candidate for pruritic dermatoses.

Proper skin hydration is essential for maintaining healthy skin. HA is the key molecule involved in skin moisture because of its unique capacity to bind up to 1000 times its weight in water. Moisturization and barrier integrity in the epidermis are largely mediated by HA, synthesized by the HAS family of enzymes (HAS‐1, HAS‐2, and HAS‐3) [40, 41]. These enzymes differ in the size of HA polymers they produce and their expression under stress or repair conditions [40, 42]. In this study, PCLP significantly upregulated the expression of all three HAS isoforms in both child and adult keratinocytes. These results suggest that PCLP may prevent loss of skin moisture.

Interestingly, pediatric keratinocytes exhibited stronger HAS‐1 and HAS‐2 responses, while HAS‐3 expression peaked at 0.250% in both age groups. These results may reflect differential regulatory dynamics between age‐specific keratinocytes and suggest that PCLP can enhance hydration by increasing HA synthesis. Previous studies have shown that increased HAS activity correlates with improved transepidermal water retention, elasticity, and recovery from barrier disruption [43, 44]. Furthermore, upregulation of HAS enzymes may also contribute to anti‐inflammatory effects, as high‐molecular‐weight HA is known to modulate immune cell activity and inhibit pro‐inflammatory cytokine release [45].

Oxidative stress is a fundamental contributor to skin inflammation, aging, and barrier impairment. LPS stimulation led to significant ROS accumulation in keratinocytes, demonstrating the model's reliability in inducing redox imbalance. PCLP consistently reduced ROS levels in both cellular ROS assay and SOD assays, confirming its antioxidant capacity. The peak effect observed at 0.250% suggests this concentration may represent an optimal balance between cellular uptake and bioactive concentration. The observed ROS‐scavenging effect may be attributed to the presence of polyphenols, flavonoids, or other active compounds capable of neutralizing free radicals or enhancing endogenous antioxidant defense systems like SOD and catalase [46, 47].

Notably, ROS suppression is closely linked with reduced activation of inflammatory pathways such as NF‐κB [48, 49], indicating that PCLP may indirectly inhibit cytokine production by restoring redox homeostasis. The parallel improvements in ROS reduction, TSLP/ET‐1 suppression, and HAS upregulation suggest a multi‐targeted mode of action, which is especially valuable in treating multifactorial skin disorders.

While both NHEK and HaCaT keratinocytes responded favorably to PCLP, pediatric cells exhibited more pronounced improvements in HAS‐1 and HAS‐2 expression, as well as slightly greater ROS suppression. This may reflect intrinsic differences in cellular metabolism, gene regulation, or antioxidant capacity between developing and mature skin. Pediatric skin is known to exhibit higher basal proliferation and different barrier lipid profiles, which could influence responsiveness to external compounds [50, 51].

To provide greater transparency and interpretability at the botanical level, we further summarized a botanical‐to‐endpoint mapping (Table S3) that links the major phytochemical classes reported for each plant, such as flavonoids, phenolic acids, and organic acids, with the biological directions observed across the TSLP, ET‐1, HAS, ROS, and SOD endpoints. This comparative framework helps to visualize how the predominant constituents of PLCP may collectively contribute to the anti‐inflammatory, anti‐pruritic, moisturizing, and antioxidant effects demonstrated in keratinocyte assays. Rather than implying single‐compound causality, this matrix is intended to inform plausibility.

The current primary treatment for atopic dermatitis is topical glucocorticoids [52]. While some experts suggest minimal side effects in children under medical supervision, the recurrence of chronic skin conditions may necessitate long‐term use of glucocorticoids and immunosuppressants, potentially leading to adverse effects [53]. Concerns over these side effects can result in poor patient adherence among parents [54]. Our study focuses on PCLP as a solution to these challenges. PCLP, a natural extract, could avoid the side effects associated with hormones and immunosuppressants. In vitro tests have confirmed PCLP's efficacy in skin moisturization, anti‐inflammatory, antioxidant, and even anti‐itch properties, particularly beneficial for pediatric patients. This innovation offers a novel approach to enhancing skin health and care, expanding insights into natural products for human health management.

While our study focused on functional endpoints with a composite extract, compound‐level fingerprinting, such as untargeted LC–MS, and causal attribution will be addressed in subsequent work to complement the present dataset.

This study demonstrates that PCLP exerts potent multifunctional effects, suppressing inflammation and itch mediators, enhancing HA‐related hydration pathways, and mitigating oxidative stress—in both pediatric and adult keratinocyte models, especially for children. These results support its potential application as a dermocosmetic or therapeutic agent for inflammatory and barrier‐compromised skin conditions. Further studies should focus on identifying the active constituents of PCLP, elucidating specific molecular targets, and validating these effects in vivo and clinical trials.

## 5 Conclusion

This study demonstrates that PCLP exerts potent, multifaceted benefits on keratinocyte health under inflammatory and oxidative stress conditions. By significantly inhibiting key pruritogenic and inflammatory mediators (TSLP and ET‐1), enhancing the expression of hyaluronic acid synthases (HAS‐1, HAS‐2, HAS‐3), and mitigating oxidative damage through ROS reduction and SOD activation, PCLP addresses the principal pathogenic mechanisms underlying atopic dermatitis and related skin disorders. The more robust effects observed in pediatric keratinocytes underscore the extract's particular relevance for children, who are especially vulnerable to long‐term corticosteroid side effects. Taken together, these data position PCLP as a compelling natural therapeutic candidate for improving skin barrier function, reducing inflammation and itch, and restoring antioxidant balance. Future work should isolate PCLP active constituents, elucidate precise molecular targets, and validate its efficacy and safety in three‐dimensional skin models and clinical trials. By offering a holistic, plant‐based alternative, PCLP holds promise for advancing pediatric dermatological care beyond conventional steroidal treatments.

## Author Contributions

C.J., H.E.K., K.W.C., and Y.‐H.L. performed the research. Y.J.C., K.W.C., P.N.C., and H.C.Y. designed the research study. Y.J., X.Z., L.T.T.L., W.H.K., J.H.C., and N.N.G. analyzed the data. C.J., P.N.C., and Y.J.C. wrote the paper.

## Funding

This research was supported by a grant of the Korea Health Technology R&D Project through the Korea Health Industry Development Institute (KHIDI), funded by the Ministry of Health and Welfare, Republic of Korea (grant number: KH129481).

## Ethics Statement

This study does not contain any studies with human participants or animals performed by any of the authors. All cell lines (NHEK and HaCaT) were anonymized commercial cell lines.

## Conflicts of Interest

Y.J.C., W.H.K., and J.H.C. are/were employees of Zero to Seven Inc. (Seoul, Republic of Korea), which is developing the product. All other authors declare no conflicts of interest.

## Supporting information


**Data S1:** jocd70613‐sup‐0001‐Supinfo.docx.

## Data Availability

The data that support the findings of this study are available from the corresponding author upon reasonable request.
