# Supplementary material for: A Natural Composite Extract Restores Skin Barrier Function by Modulating Inflammatory, Hydration, and Redox Pathways
Source: J Cosmet Dermatol. 2025 Dec 13;24(12):e70613. doi: 10.1111/jocd.70613 (PMC12701548; doi:10.1111/jocd.70613)
Supplement: Supplementary file 1 — Data S1: jocd70613‐sup‐0001‐Supinfo.docx. [file JOCD-24-e70613-s001.docx]

**Supplementary table S1.** QC summary of total polyphenols and DPPH.

| Parameter | Extraction type | Mean ± SD | Relative difference |
| --- | --- | --- | --- |
| Total polyphenol content (µg GAE/g extract) | VE extraction | 497.83 ± 5.21 | +43.7% vs. general |
|  | General extraction | 346.60 ± 4.78 |  |
| DPPH radical scavenging activity (IC₅₀, µg/mL) | VE extraction | 182.4 ± 3.2 | –35.5% vs. general |
|  | General extraction | 283.0 ± 6.5 |  |

**Supplementary table S2**. Composition of PLCP

| Component (INCI name) | CAS No. | Concentration range (% w/w) |
| --- | --- | --- |
| Water | 7732-18-5 | 80.00 – 90.00 |
| 1,2-Hexanediol | 6920-22-5 | 5.00 – 15.00 |
| Phyllostachys bambusoides extract | — | 2.00 – 8.00 |
| Chrysanthemum indicum flower extract | — | 0.20 – 0.80 |
| Lonicera japonica (honeysuckle) flower extract | — | 0.20 – 0.80 |
| Prunus mume flower extract | — | 0.02 – 0.08 |

**Supplementary table S3**. Botanical-to-endpoint contribution matrix.

| Botanical source | Major reported phytochemical classes | Likely biological contributions based on literature | Correlated experimental endpoints in this study |
| --- | --- | --- | --- |
| *Phyllostachys bambusoides* | Phenolic acids, flavonoids, lignans, polysaccharides [1-3] | Antioxidant, ROS scavenging, anti-inflammatory, moisturizing, barrier-supporting, [4, 5] | ↓ TSLP, ↓ ROS, ↑ SOD, ↑ HAS3 |
| *Lonicera japonica* | Chlorogenic acid, luteolin derivatives [6] | Anti-inflammatory, anti-itch [7, 8] | ↓ TSLP, ↓ ET-1 |
| *Chrysanthemum indicum* | Luteolin, apigenin, caffeic acid [9] | Antioxidant, anti-inflammatory, soothing [10, 11] | ↓ TSLP, ↓ ROS, ↑ SOD |
| *Prunus mume* | Organic acids (citric, malic), flavonoids [12] | Mild antioxidant, skin-soothing [13] | ↑ HAS2, ↓ ROS |

**Supplementary reference**

1. Cheng Y, Wan S, Yao L, Lin D, Wu T, Chen Y, et al. Bamboo leaf: A review of traditional medicinal property, phytochemistry, pharmacology, and purification technology. J Ethnopharmacol. 2023;306:116166.

2. Li HX, Phong NV, Lim SD, Kim YH, Li W, Yang SY. Bioactive constituents isolated from the leaves of Phyllostachys Bambusoides with potent soluble epoxide hydrolase inhibitory activity: enzyme kinetics, molecular docking, and molecular dynamics simulations. J Comput Aided Mol Des. 2025;39(2):100.

3. Gong J, Xia D, Huang J, Ge Q, Mao J, Liu S, et al. Functional components of bamboo shavings and bamboo leaf extracts and their antioxidant activities in vitro. J Med Food. 2015;18(4):453-9.

4. Kumar S, Sharma G, Sidiq T, Khajuria A, Jain M, Bhagwat D, et al. Immunomodulatory potential of a bioactive fraction from the leaves of Phyllostachys bambusoides (bamboo) in BALB/c mice. Excli j. 2014;13:137-50.

5. Qi XF, Kim DH, Yoon YS, Li JH, Jin D, Deung YK, et al. Effects of Bambusae caulis in Liquamen on the development of atopic dermatitis-like skin lesions in hairless mice. J Ethnopharmacol. 2009;123(2):195-200.

6. Shang X, Pan H, Li M, Miao X, Ding H. Lonicera japonica Thunb.: ethnopharmacology, phytochemistry and pharmacology of an important traditional Chinese medicine. J Ethnopharmacol. 2011;138(1):1-21.

7. Bai X, Rao X, Wang Y, Shen H, Jin X. A homogeneous Lonicera japonica polysaccharide alleviates atopic dermatitis by promoting Nrf2 activation and NLRP3 inflammasome degradation via p62. J Ethnopharmacol. 2023;309:116344.

8. Bai X, Liu P, Shen H, Zhang Q, Zhang T, Jin X. Water-extracted Lonicera japonica polysaccharide attenuates allergic rhinitis by regulating NLRP3-IL-17 signaling axis. Carbohydr Polym. 2022;297:120053.

9. Shao Y, Sun Y, Li D, Chen Y. Chrysanthemum indicum L.: A Comprehensive Review of its Botany, Phytochemistry and Pharmacology. Am J Chin Med. 2020;48(4):871-97.

10. Sun S, Jiang P, Su W, Xiang Y, Li J, Zeng L, et al. Wild chrysanthemum extract prevents UVB radiation-induced acute cell death and photoaging. Cytotechnology. 2016;68(2):229-40.

11. Lee DY, Choi G, Yoon T, Cheon MS, Choo BK, Kim HK. Anti-inflammatory activity of Chrysanthemum indicum extract in acute and chronic cutaneous inflammation. J Ethnopharmacol. 2009;123(1):149-54.

12. Nakamura S, Fujimoto K, Matsumoto T, Nakashima S, Ohta T, Ogawa K, et al. Acylated sucroses and acylated quinic acids analogs from the flower buds of Prunus mume and their inhibitory effect on melanogenesis. Phytochemistry. 2013;92:128-36.

13. Matsuda H, Morikawa T, Ishiwada T, Managi H, Kagawa M, Higashi Y, et al. Medicinal flowers. VIII. Radical scavenging constituents from the flowers of Prunus mume: structure of prunose III. Chem Pharm Bull (Tokyo). 2003;51(4):440-3.
